# Xanomeline-trospium: defining its place among the current antipsychotic landscape

**DOI:** 10.1017/neu.2025.10032

**Published:** 2025-09-04

**Authors:** Sajitha Nair, Sukhi Shergill, Eromona Whiskey

**Affiliations:** 1 Kent and Medway NHS Partnership Trust, Maidstone, UK; 2 Pharmacy Department, South London & Maudsley NHS Foundation Trust, London, UK; 3 Institute of Pharmaceutical Science, King’s College London, London, UK; 4 Kent and Medway Medical School, Canterbury, UK; 5 University of Kent, Canterbury, UK; 6 Institute of Psychiatry, Psychology and Neuroscience, King’s College, London, UK

**Keywords:** Schizophrenia, cobenfy, xanomeline-trospium, antipsychotic, muscarinic receptors

## Abstract

Progress in the development of new and improved medications for psychosis has been notably slow and disappointing. The first treatment for schizophrenia was introduced in early 1950s and the majority of medications available today exclusively function through dopamine antagonism. The search for a new drug treatment with a different mechanism of action was extremely slow-paced mainly due to the limited understanding of the aetiology, pathophysiology and genetics of schizophrenia. Given the fact that a third of people do not respond to dopamine antagonists, there is a clear need for an antipsychotic with a different mechanism of action. In 2024, FDA approved a new medication for psychosis branded as Cobenfy. This xanomeline-trospium combination works via cholinergic pathway and the dual M1 and M4 receptor activation helps regulates dopaminergic and glutaminergic neurotransmission as well, thereby restoring balance in these circuits. Acetylcholine also helps improve cognitive processing including attention, learning and sensory gating. In this article, we try to understand the place of this unique drug in the antipsychotic ladder. We also explore the clinical scenarios where this medication can be effective as well as the potential future outlook when it comes to the treatment of schizophrenia.

## Graphical abstract

**Figure:**
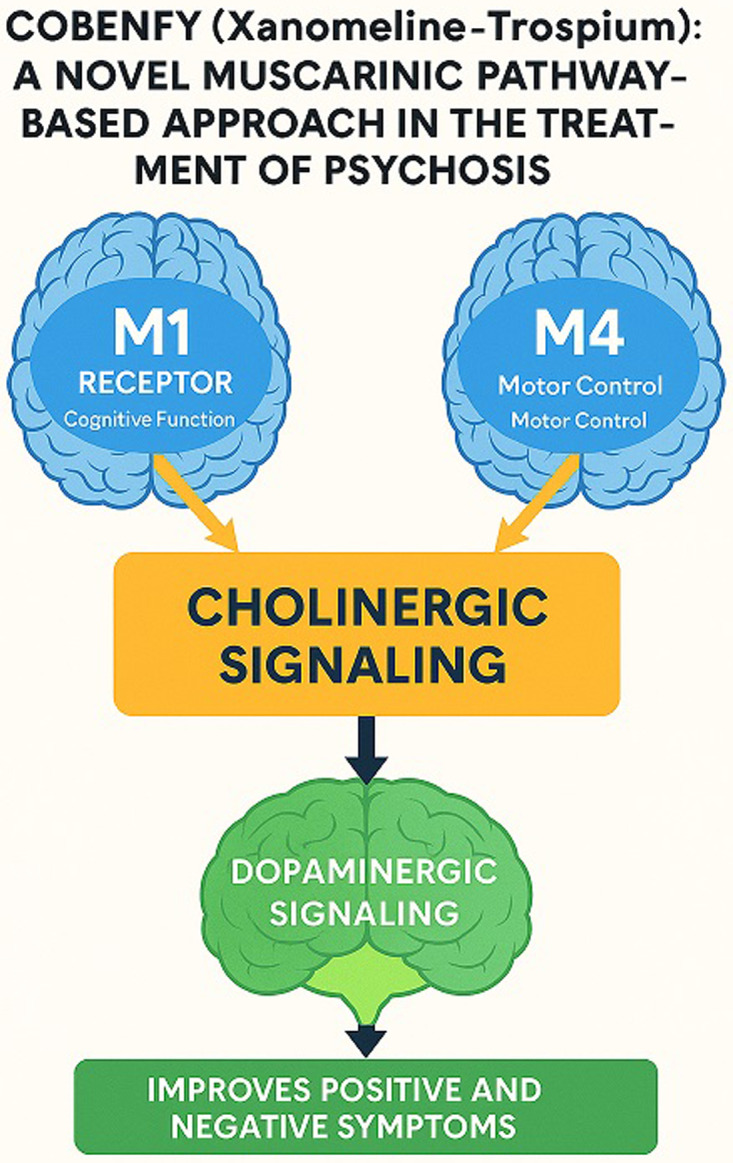

**Table:** 

Summation	Description
1. Xanomeline/trospium offers a distinct therapeutic beyond dopamine receptor targeting in the treatment of schizophrenia.	By selectively targeting M1/M4 receptors, it offers a unique mechanism that improves positive, negative and cognitive symptoms while avoiding dopamine-blockade-related side effects
2. This combination may suit patients poorly responsive or intolerant to traditional antipsychotics	It shows promise in individuals who experience significant side effects like extrapyramidal side effects, tardive dyskinesia, or metabolic concerns or inadequate response from standard dopamine-blocking medications
3. Xanomeline’s cognitive benefits stem from its enhancement of cholinergic and glutaminergic signalling.	M1 receptor activation promotes better brain connectivity, attention and memory by strengthening neuroplasticity and synaptic functions
4. Current data positions xanomeline/trospium as a secondary treatment option pending broader clinical validation	While innovative and promising, its role will depend on further real-world evidence, particularly for long-term efficacy and comparisons with agents like clozapine.

**Table:** 

Perspective	Details
1. Efficacy remains moderate and comparative advantage unproven.	Available research showed no significant differences in short-term efficacy compared to existing treatments like olanzapine or risperidone.
2. Limited data on sustained outcomes including long-term safety and clinical utility.	Despite encouraging early trial results, its long-term effect on cognitive symptoms, functional improvement, and treatment- resistant schizophrenia is not yet sustained
3. Practical barriers may restrict broader use	Side effects, cost concerns, and limited availability restrict its use, especially in resource-constrained settings.

## Introduction

Schizophrenia is a complex and chronic mental illness that profoundly disrupts various domains of an individual’s functioning. It is primarily characterised by disturbance in thought processes, perception, emotions, cognition and social interactions. Individuals affected with schizophrenia often experience hallucinations, delusions, disorganised thinking, emotional flattening, and significant cognitive impairments. These symptoms are typically grouped into three categories: positive symptoms (such as hallucinations and delusions), negative symptoms (such as apathy and social withdrawal), and cognitive symptoms (such as impaired concentration and problem solving skills). Collectively, these disturbances interfere with a person’s ability to perceive reality accurately, manage relationships, and perform daily tasks, often resulting in profound personal and social impairment. It is indeed considered as one of the most disabling psychiatric disorders worldwide.

Reference to the condition date back as early as 1500 BCE with more detailed descriptions emerging in the 19^th^ century. However, it was not until the 1950s that the ‘dopamine hypothesis’ of schizophrenia was introduced (Jablensky, 2010). More than two decades later, in late 1970s and early 1980s, the ‘glutamate hypothesis’ was proposed (Moghaddam & Javitt, 2012). Subsequent research has confirmed that schizophrenia is influenced by a complex interplay of multiple neurotransmitters, including dopamine, glutamate, GABA, acetylcholine and serotonin, all contributing to its diverse symptoms (Bansal & Chatterjee, 2021).

It is now well established that schizophrenia involves a multifaceted pathophysiology encompassing genetics, neurotransmitter dysregulations, and neuroanatomical abnormalities (Luvsannyam *et al.,* 2022). However, this understanding has not directly led to the development of treatments targeting the different dysregulations. Up until recently, we only had dopamine antagonism as the primary antipsychotic action.

### Current antipsychotics

Positive symptoms of schizophrenia arise from excessive dopamine release with excessive dopamine receptor sensitivity, faulty glutamate signalling and reduced GABAergic inhibition, while negative symptoms result from decreased dopamine levels and reduced glutamate transmission in the prefrontal cortex.

First-generation or typical antipsychotics act through non-selective dopamine antagonism, primarily targeting D2 receptors and thus highly effective for positive symptoms. This non-selective dopamine blockade also contributes to a wide range of side effects including extra-pyramidal side effects (EPSE’s), hyperprolactinaemia, cholinergic side effects, and side effects related to histaminergic blockade (Wubeshet *et al.,* 2019).

Second-generation or atypical antipsychotics block D2 receptors and also serotonin 5-HT2A receptors (Serretti *et al.,* 2004). The most effective medication in this group is clozapine which remains the gold standard for treatment-resistant schizophrenia (Serretti *et al.,* 2004). These atypical medications do not belong to a single neurochemical category, rather, they represent a heterogeneous group with diverse effects on neurotransmission. For example, olanzapine is a potent dopamine and serotonin receptor antagonist, amisulpride selectively blocks D2/D3 receptors while exhibiting dopamine-enhancing or antagonistic effects depending on the dose and aripiprazole functions as a partial dopamine agonist (Serretti *et al.,* 2004).

The atypical antipsychotics improve positive symptoms of schizophrenia but the impact on negative and cognitive symptoms is limited. An exception to this is cariprazine, which has been shown to improve negative symptoms of schizophrenia (Németh *et al*., 2021). Despite a lower risk of EPSE’s when compared to typical antipsychotics, they still carry potential drawbacks, including sedation, metabolic syndrome, and residual motor movement risks.

### Challenges in developing newer antipsychotics

Advancements in understanding the complex neurochemical mechanisms underlying schizophrenia, along with the side-effect profiles of existing treatments, have fuelled interest in developing novel antipsychotics with unique mechanisms of action. Some of the novel approaches include medication like lumateperone, with a unique dopaminergic profile of post-synaptic dopamine receptor antagonist with a pre-synaptic D2 receptor partial agonism, thus preserving striatal dopaminergic function and minimising motor side effects (Begni *et al.,* 2025). Pimavanserin is another example and it is a potent serotonin 5 HTA-2 inverse agonist rather than antagonist with minimal impact on dopamine receptors and is approved for treating psychosis in Parkinson’s disease, where serotonin dysregulation is believed to play a key role (Weintraub *et al.,* 2024). It was later investigated as add-on therapy for schizophrenia, however a phase III study failed to show any benefit and hence it was discontinued (Begni *et al.,* 2025).

But for every success, there are countless failures, far outnumbering the instances of success and it has been more than 70 years that a medication with a novel mechanism of action has been launched. . For example, pomaglumetad methionil is a drug which activates group II metabotropic glutamate receptors, specifically mGluR2 and mGluR3 but failed in trials (Begni *et al.,* 2025). Similarly, iclepertin is a glycine transporter inhibitor which was mainly studied for its cognitive improvement properties, however this too failed the trials (Begni *et al.,* 2025). Ulotaront is an agonist for trace amine-associated receptor 1 (TAAR1) as well as for serotonin 5-HT1A receptors and sadly this too could not demonstrate significant benefit (Begni *et al.,* 2025).

The primary challenge lies not only in controlling positive symptoms but also in effectively addressing negative symptoms and cognitive impairment, both of which still remain the most difficult aspects of schizophrenia to treat. Unlike positive symptoms, negative symptoms are more enduring and multifactorial and the gains may be subtle, hard to measure or masked by broader functional impairments. Additional obstacles include the need to balance efficacy with side effects, and the uncertainty surrounding treatment resistance in certain patient populations. Moreover, antipsychotic drug development is a lengthy, costly and high-risk process, often limited by inadequate preclinical testing, the necessity for large-scale clinical trials, and regulatory hurdles. Pharmaceutical companies are often reluctant to invest in psychotropic drug development due to high failure rates, lower profitability and stringent approval processes (Seth *et al.,* 2022).

Despite these challenges, several novel antipsychotics are currently in development including glutamate receptor modulators, monoamine receptor modulators, acetylcholine receptor modulators, cannabinoid receptor modulators and mixed receptor modulators (Biso *et al.,* 2025).

## Discussion

### History of xanomeline/trospium development

In September 2024, the FDA approved the combination of xanomeline and trospium, now branded as Cobenfy, for the treatment of Schizophrenia. This approval was based on the results of a successful five-week Phase 2 trial and two Phase 3 trials --- EMERGENT-1, EMERGENT-2, and EMERGENT-3 (Kaul *et al.,* 2024). The trials proved that xanomeline-trospium combination was efficacious and well tolerated for patients with schizophrenia (Kaul *et al.,* 2024)

Xanomeline was initially studied in the 1990s as a potential treatment for Alzheimer’s disease. In patients with Alzheimer’s disease, xanomeline not only showed a positive trend towards cognitive improvement, but also led to an unexpected reduction in behavioural disturbances, including hallucinations, vocal outbursts, and agitation (Bodick *et al.,* 1997). However, due to significant peripheral cholinergic side effects and a narrow therapeutic index, its development was discontinued.

Interest in xanomeline was revived after 2012, particularly when researchers found that adding trospium could help mitigate its peripheral side effects. Trospium, a well-established non-selective peripheral antimuscarinic agent, does not cross the blood-brain barrier, allowing it to counteract peripheral cholinergic side effects without interfering with xanomeline’s central actions.

### Mechanism of action

**Figure 1.**
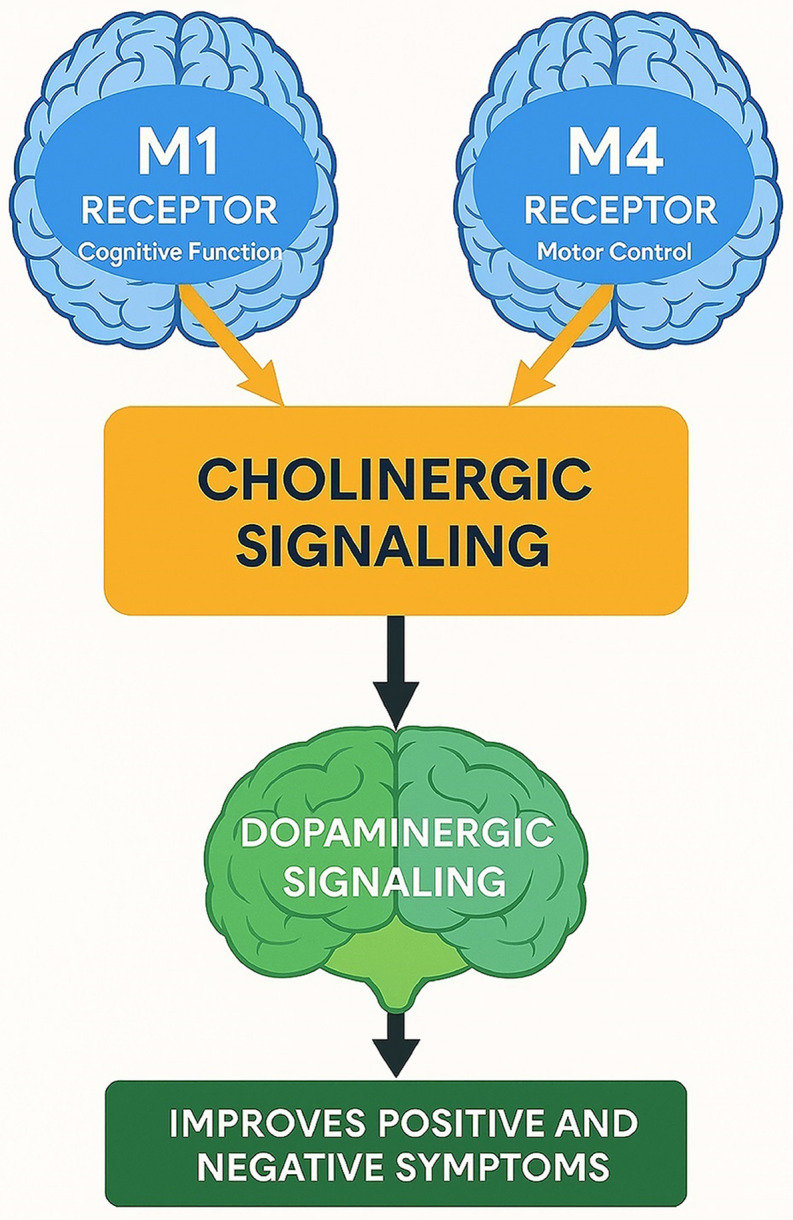
Cholinergic modulation of dopaminergic pathways via M1 and M4 receptors in schizophrenia. This diagram depicts how M1 receptors (linked to cognitive functions) and M4 receptors (associated with motor control) activate cholinergic signalling, which in turn modulates dopaminergic pathways in the brain. Enhanced dopaminergic signalling contributes to the reduction of both positive and negative symptoms in schizophrenia. Brain icons represent key neural targets influenced by receptor-specific activity.

Cholinergic transmission in the striatum is vital in regulating synaptic plasticity, neuronal excitability, synaptic strength and the formation of dendritic spines and synapses – all of which are essential for memory, attention and other cognitive functions (Drever *et al.,* 2011). Xanomeline acts as an M1/M4 dual muscarinic receptor agonist, stimulating acetylcholine release (Shin *et al.,* 2015). It primarily targets M1 and M4 receptors located in the cortex, striatum, and hippocampus, playing a crucial role in cognition and emotional regulation and modulation of psychotic and behavioural disturbances (Figure 1).

M1 receptors are predominantly post-synaptic and enhances acetylcholine responsiveness through the Gq/11 signalling pathway. In the healthy brain, M1 activation supports cholinergic tone, by enhancing glutamate transmission, which may then feedback to increase cholinergic activity and this then supports cognitive circuits as well. In schizophrenia, M1 receptor dysfunction leads to reduced cholinergic tone which impairs glutamate signalling (Ghoshal *et al.,* 2016). This disrupted glutamate activity contributes to reduced dopamine in the prefrontal cortex, causing negative and cognitive symptoms, and dysregulated dopamine in the striatum, contributing to positive symptoms. Xanomeline selectively enhances M1 in the cortex and striatum and this contributes to the reduction in positive, negative and cognitive symptoms (Yohn *et al.,* 2022).

M4 receptors are primarily expressed on pre-synaptic terminals and act as auto-receptors that reduce acetylcholine through the Gi/o signalling pathway (Fu *et al.,* 2024). They help control excessive dopamine in the mesolimbic pathway by reducing acetylcholine, thus reducing positive symptoms while minimising motor side effects by reducing the excessive cholinergic tone (Fu *et al.,* 2024). M4 activation also stabilises cognition by reducing glutamate transmission.

Xanomeline improves cognitive function primarily through selective M1 receptor activation. M1 receptors have been shown to modulate functional connectivity within the brain, enhancing cognitive and sensory processing circuits (Montani *et al.,* 2021). M1 receptor has been shown to enhance neuronal excitability and synaptic plasticity in rodent brain (Montani *et al.,* 2021). Apart from this M1 receptor amplifies the downstream effects of cholinergic signalling which are essential for attention and memory encoding, and restores glutamate and dopamine signalling, thereby improving executive functions. M1 receptors has been linked to greater synchrony of gamma oscillations, and this can then improve cognitive deficits linked to schizophrenia (Etterton *et al.,* 2017). Lastly, xanomeline via reduction of positive symptoms allows better focus and engagement.

Xanomeline influences gene expression, including the activation of immediate early genes like c-fos, promoting synaptic strengthening and neuroplasticity (Perry *et al.,* 2001). It also upregulates genes associated with neuroplasticity and cell survival, such as c-fos, sirtuin-1, and haem oxygenase-1. This gene expression enhances long-term potentiation, synaptic strengthening, and cognitive resilience.

An important point to note would be that xanomeline does not have a direct action on post-synaptic dopamine receptors like typical antipsychotics. Instead, its pre-synaptic modulation offers a more refined and potentially superior approach by fine-tuning dopamine release rather than completely inhibiting it.

### Evidence supporting the effectiveness of cholinergic modulation

Early results from the EMERGENT-4 and EMERGENT-5 open-label trials, conducted over 52-weeks, have reported sustained improvement along with enhanced quality of life across physical, social, emotional and role-functioning domains with the use of xanomeline/trospium in Schizophrenia (Kaul *et al.,* 2025).

One line of the evidence comes from clozapine which is known to act as a muscarinic acetylcholine receptor (mAChR) antagonist, or possibly as a weak partial agonist. However, a notable distinction is that norclozapine, clozapine’s active metabolite, is a potent agonist of mAChRs, particularly at the M1 receptor subtype. No other currently used antipsychotics or their metabolites are known to function as mAChR agonists, suggesting that this agonist activity of norclozapine may be one of the key properties that differentiates clozapine from other atypical antipsychotics.

Scarr *et al*. (2009) reported reduced M1 receptor density in the prefrontal cortex and hippocampus in about 25–50% of a subset of patients with schizophrenia indicating a possible subset of patients with a cholinergic deficit (Scarr *et al.,* 2009). Similarly, neuroimaging studies including PET scans have indicated diminished cholinergic function in patients with schizophrenia and have linked this to the presence of cognitive symptoms.

A link between the nicotinic system and schizophrenia is also supported by the strikingly high prevalence of tobacco dependence in individuals with schizophrenia, with around 65% of patients being smokers. Cigarette smoking improves cognitive functions (although temporarily) and hence it is assumed that patients with schizophrenia use this as a self-medication strategy. Lastly, it is well known that a higher anticholinergic burden in schizophrenia is associated with worsened cognitive and psychotic symptoms, highlighting the potential relevance of cholinergic modulation in the treatment of schizophrenia, although its precise therapeutic role remains to be fully clarified.

A Phase 3 ARISE trial conducted by Bristol Myers Squibb (BMS) evaluated xanomeline/trospium, and even though xanomeline/trospium demonstrated a 2.0 point greater reduction in PANSS total score compared to placebo; the difference did not reach statistical significance (**ARISE** clinical **trial** (KAR-012), 2025). In the same trial, a post-hoc subgroup analysis revealed that patients receiving non-risperidone antipsychotics (such as paliperidone, aripiprazole, ziprasidone, lurasidone and cariprazine) experienced a statistically significant greater reduction in PANSS total score when treated with Cobenfy compared to placebo (−15.1 vs. −11.7; *P* = 0.03) (24). In contrast, no significant difference was observed in patients receiving risperidone as their background therapy (**ARISE** clinical **trial** (KAR-012), 2025). It is interesting to note the weaker effects of xanomeline/trospium in patients taking risperidone in this trial. This could be due to the fact that risperidone is a strong dopaminergic and serotonergic antagonist with negligible muscarinic action, leading to a maximum dopaminergic modulation, leaving less room for xanomeline to provide additional benefit. It could also be that high dopamine blockade might mask or interfere with the indirect dopamine modulation from xanomeline’s M4 activation. Lastly it may be possible that the patients prescribed risperidone may have a different illness profile, severity or symptom type, which might influence how they respond to adjunctive treatments.

Although the ARISE trial did not meet its primary efficacy end point, the observed numerical improvements observed in some subgroups highlight the need for further exploratory and confirmatory studies to assess potential differential responses. It may be important to keep in mind at this stage that early failure does not doom a drug’s future and real-world effectiveness often reveals benefits that early endpoints miss. Several antipsychotics that are widely used today including clozapine, aripiprazole, quetiapine etc. have had disappointing or inconclusive results in early trials but later became essential treatments due to refined trial design or better understanding of patient subgroups or in terms of long-term safety profiles.

## Strengths and limitations

### Strengths

1. Lower risk of movement disorders including EPSE’s and tardive dyskinesia
2. Trials suggest that xanomeline/trospium improves performance on a cognitive outcome measure in the subgroup of participants with clinically significant baseline cognitive impairment, however long-term trials will be needed to substantiate such findings.
3. Minimal impact on weight gain and metabolic disturbances compared to other antipsychotics.
4. Possibly enhances motivation and social interaction by boosting cholinergic activity in the prefrontal cortex, long-term trials needed to ascertain this.

### Limitations

1. Cholinergic side effects
2. Limited long-term data on both safety and efficacy
3. Higher cost and potential accessibility challenges
4. Evidence so far suggests Cobenfy has moderate efficacy and currently we lack studies citing its use in treatment-refractory cases or comparing its efficacy with clozapine. In one of the network meta-analysis review which compares Cobenfy to olanzapine, risperidone and aripiprazole, the conclusion was there were no clear differences in short-term efficacy among the active interventions (Wright *et al.,* 2024).
5. With the above evidence, xanomeline/trospium may need to be used alongside other antipsychotics to achieve full symptom control, which could increase the overall antipsychotic burden (Table 1).

### Role in the antipsychotic landscape

Currently, Cobenfy occupies a distinct position among antipsychotic treatments due to its unique mechanism of action. While it may indirectly involve the dopamine pathway, its impact is far more targeted and selective compared to traditional dopamine-blocking agents. Cobenfy may offer potential benefits for certain patients who are unable to tolerate conventional antipsychotics. However, at this stage the clinical data remains preliminary and require further validation.

### Potential candidates for xanomeline/trospium

Identifying the ideal patient population for Cobenfy remains challenging, but certain groups may benefit the most:

1. Patients intolerant to dopamine-based antipsychotics or those who have experienced intolerable EPSE’s, tardive dyskinesia or hyperprolactinaemia with previous treatments.
2. Individuals with metabolic syndrome, weight concerns or at a high risk of developing metabolic side effects.
3. Patients struggling with cognitive impairment or negative symptoms that standard antipsychotics fail to address effectively. There is evidence that cognitive improvement can reduce psychotic vulnerability and can improve medication response and functional improvement (Keshavan & Eack, 2023).
4. Those seeking an alternative mechanism of action or adjunctive treatment, especially partial responders or individuals with treatment-resistant schizophrenia. It may be too early to consider Cobenfy as a solo agent for treatment-resistant cases, but it can be promising as an add-on therapy in these patients with a careful case by case evaluation.

### Clinical decision-making and future outlook

Clinicians must carefully assess the risk-benefit ratio before transitioning patients to xanomeline/trospium. Ideally, treatment decisions should be shared between clinicians and patients, considering factors such as symptom severity, current treatment efficacy, side effects, risk tolerance, and personal preference.

At present, xanomeline/trospium may be best positioned as a mid-tier option in the antipsychotic treatment hierarchy. It can be considered after the failure of 1-2 standard antipsychotics but before the label of treatment resistance comes into picture. However, with further clinical experience and real-world studies, its role could evolve into a first-line option for specific patient subgroups.

Xanomeline/trospium may not be suitable for patients prone to gastrointestinal side effects or those with pre-existing gastrointestinal conditions, as it would be difficult for them to tolerate the medication. Additionally, switching stable patients from their current dopamine-based antipsychotics may not be advisable especially if they are experiencing good symptom control and minimal side effects. While Cobenfy presents a new therapeutic option which often encourages people to have a trial, at present the data suggests it does not offer a significant response in treatment-resistant patients.

Future research interest for xanomeline/trospium combination could involve studying the long-term efficacy and safety, studying the effect on cognitive symptoms associated with schizophrenia in the long term, it’s use in other neuropsychiatric or neurodegenerative conditions, possibly looking into development of alternative formulations including long acting injections and mechanistic studies to fully understand the cholinergic pathway and its involvement in psychosis. Exploring the combination therapy with the current available antipsychotics may also be an interesting research option, especially given the findings of the ARISE trial (ARISE clinical trial (KAR-012), 2025).

Given the modest and statistically non-significant results in the ARISE trial, it is important to interpret the current findings with caution. While post-hoc subgroup analysis hint at a differential effect, depending on background antipsychotic use, these findings require replication. At this point, it remains uncertain whether further trials will be pursued by BMS or whether other muscarinic targeting compounds will yield more robust outcomes. Until then the clinical enthusiasm for xanomeline/trospium should be tempered by the need for stronger confirmatory evidence.

## Conclusion

In conclusion, currently xanomeline/trospium is not intended to replace traditional antipsychotics entirely, but rather to serve as a much-needed alternative for certain subsets of patients. While its precise clinical role remains to be established, xanomeline/trospium represents a promising development that broadens the therapeutic landscape for schizophrenia, particularly given its novel mechanism of action. Further studies are needed to confirm its long-term efficacy and clinical utility.

**Table 1.** Strengths and limitations of xanomeline/trospium combination

Strengths	Limitations
Reduced risk of movement related side effects	Cholinergic side effects may occur
May enhance motivation, cognition and social functioning	Long-term safety and effectiveness are uncertain
Minimal impact on weight and metabolic health	Moderate efficacy, not yet validated for treatment-resistant schizophrenia
Provides cognitive benefit in patients with baseline deficits	Higher cost and accessibility challenges
